# Knowledge and management of Münchausen’s Syndrome by proxy: a survey conducted through the compilation of a questionnaire by pediatricians belonging to the Italian Society of Pediatrics

**DOI:** 10.1186/s13052-025-01878-z

**Published:** 2025-02-25

**Authors:** Rosaria Nardello, Giada Cordova, Corinne La Spina, Ettore Piro, Gregorio Serra, Giovanni Corsello, Antonina Argo

**Affiliations:** Department of Health Promotion, Mother and Child Care, Internal Medicine and Medical Specialities “G. D’Alessandro”, University of Palermo, Palermo, Italy

**Keywords:** Münchausen Syndrome by proxy, Child abuse, Malteatred children

## Abstract

**Background:**

Munchausen syndrome by proxy represent forms of abuse with long-term psychiatric outcomes. Since the prevalence of Munchausen Syndrome by proxy is uncertain and underestimated, this study aimed to investigate and analyze the phenomenon through the compilation of an anonymous questionnaire that explores the knowledge of the phenomenon and above all its management.

**Methods:**

the study was conducted by sending an anonymous questionnaire to pediatricians who are part of the Italian Society of Pediatrics. The questionnaire consists of 18 multiple choice questions and was completed by 511 professionals.

**Results:**

The main results highlighted that the majority of doctors knows Münchausen Syndrome by proxy. However, when there is a strong suspicion of the syndrome, they mostly seek discussion with the parent or with another specialist instead of referring to the competent authorities.

**Conclusions:**

starting from the consideration that timely diagnosis is fundamental for the protection of the child, we emphasize the urgency of enhancing the recognition and management of Munchausen Syndrome by Proxy. Early diagnosis, appropriate reporting, and collaboration with child protection authorities are essential in safeguarding the well-being of vulnerable individuals.

## Background

Munchausen syndrome was first described in 1951 by Asher to define individuals who deliberately produce symptoms and signs who tend to seek medical or hospital care [1]. In 1977, Meadow adopted the term “Munchausen Syndrome by Proxy” (MBP) to describe children whose caregivers create histories of illness to their offspring and who support such histories by build physical signs and symptoms, or even by modify laboratory tests [2, 3]. The perpetrator are generally the mothers that are classified into three categories: active inducers, help seekers, and doctor addicts. Active inducers overemphasize the illnesses of their children; help seekers manipulate the children get round problems such as domestic violence and disfatisfied marriage; doctor addicts are overly suspicious [4–6].

MBP can present itself through various clinical cases including a form of systemic lupus erythematosus, induction of coma through the administration of benzodiazepines, catatonia, persistent hyperinsulinemic hypoglycemia, Avoidant Restrictive Food Intake Disorder [7–11]. MBP configures a child abuse. In particular, when considering the victim, the more appropriate term may be “pediatric condition falsification” or “medical child abuse“ [12–15]. The prevalence of MBP is uncertain and underestimated. It seems that there is no prevalence in the sex of the victim, however when the perpetrator is the mother, both sons and daughters are affected. However, when the abuser is the father, male children will be favoured. As regards the average age of diagnosis, this is around 48.6 months. Very young children tend to be affected because they are unable to defend themselves [16]. The incidence is around 0.4/100,000 children aged 2 to 16 years and 2/100,000 children under 12 months [17]. Given that the prevalence is underestimated and the great variability of the symptoms, we believe that pediatricians must know the problem to protect young patients but that they must also have the tools to act on how to behave to protect children. Furthermore, there is not always adequate awareness among health workers of the need to communicate suspected cases to the judicial authorities, and of the method of maintaining any evidence to testify to abuse in Court [14, 15].

For this reason, after having developed a questionnaire intended to explore these areas of interest, we decided to send an anonymous questionnaire to all pediatricians registered with the Italian Society of Paediatrics.

## Materials and methods

The identification of the areas of exploration of this research topic, to prepare the questionnaire, was developed by a group of doctors of different specializations (paediatricians, child neuropsychiatrists, forensic doctors, urgencies and emergencies) of Policlinic Hospital, by using an multidisciplinary approach and convergence to selected items. An expert panel of paediatricians at regional level agreed with the questionnaire items, before presenting to Ethic Committee.

The study conducted involved the compilation of an anonymous questionnaire, where the professional must indicate their gender and profession. The questionnaire is made up of 11 questions, some of which are divided into sub-questions, for a total of 18 multiple choice questions.

The questionnaire was sent to the Italian Society of Paediatrics and was completed by 511 professionals divided into: Freelance professionals, Neonatologists, Free choice paediatricians, Hospital paediatricians, Pensioners, Doctors in Training and University Teachers.

The objective of our study was to verify the knowledge of MBP among the first professionals who are in contact with children, i.e. paediatricians, at various ages of development and above all to verify the management of cases in which there is a diagnosis of suspicion and/or a certainty. This study was approved by the ethics committee Palermo 1 of “Paolo Giaccone” University Hospital of Palermo, Italy. (Table 1)

**Table 1:** Description of the various professional figures who responded to the questionnaire

Profession	*n*°	%
Freelance professionals	17	3,33
Neonatologists	51	10,00
Free choice paediatricians	219	42,94
Hospital paediatricians	198	38,82
Pensioners	4	0,78
Doctors in Training	13	2,55
University Teachers	8	1,57

## Results

1. The *fact of resorting so frequently to the doctor/first aid was*, *in your opinion*, *due to: Excessive parental apprehension; particular severity of the child’s health condition; other causes.*

From the analysis of the answers to this question, it was possible to find that 89.35% of the health professionals believed that such frequent recourse to the doctor/first aid was due to excessive parental apprehension. The main health professionals who supported this answer were free choice paediatricians and hospital paediatricians. 12% of the health professionals answered *‘particularly serious health condition of the child’*, these were all doctors in training.

2. *The parent takes the child to the doctor diagnosing diseases that are particularly rare*, *strange or with symptoms that cannot be detected by clinical investigations: a)never; b)sometimes; c) often*.

**Table 2:** Answers to question number 2

	a	b	c
Answers	104	374	30
%	20,47	73,62	5,91

Legend: a)never; b)sometimes; c) often

**Fig. 1:**
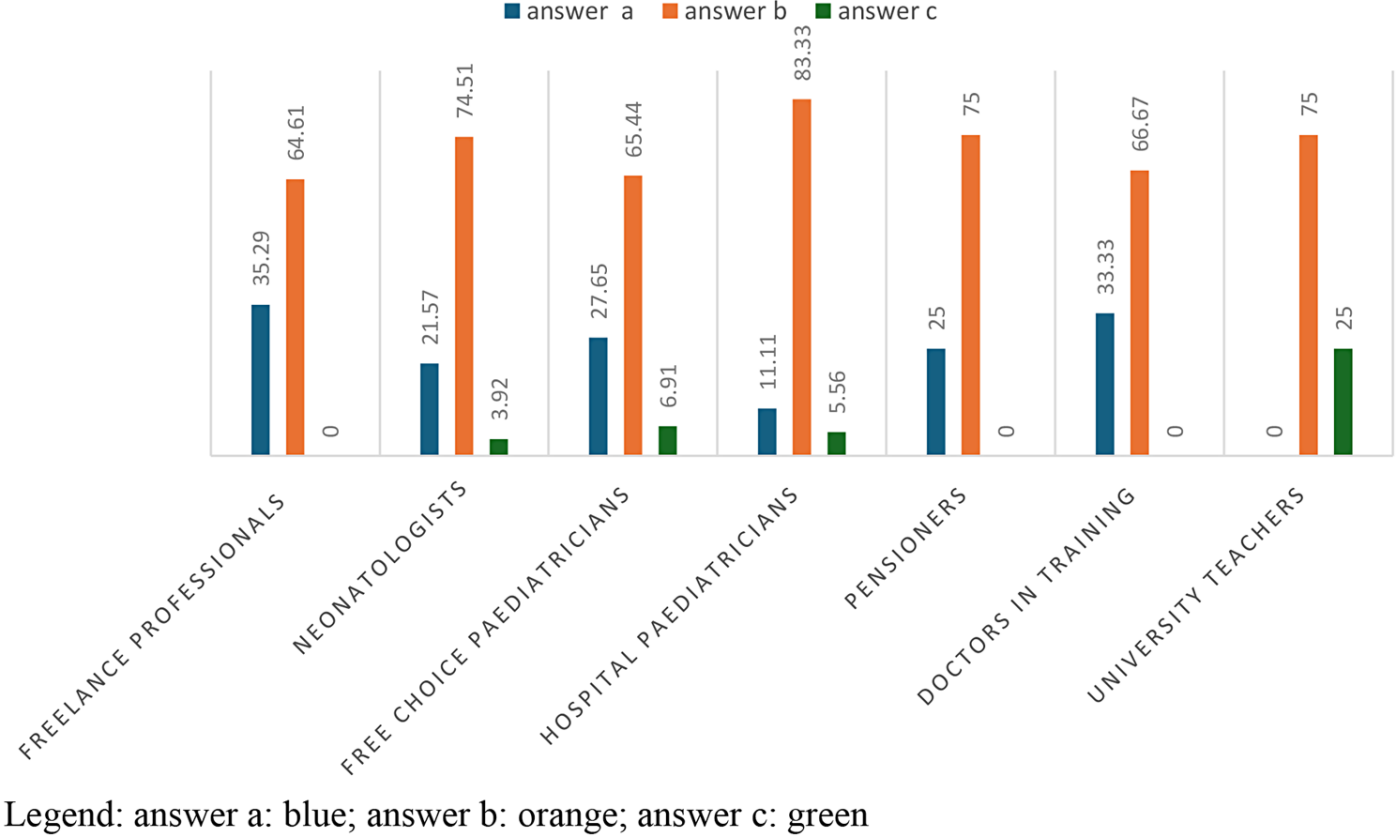
answers to question 2 divided according to profession

In Table 2, with a p-value = 0,002104, the 73,62% of healthcare professionals ansewered “sometimes”; in particular 74% of neonatologists and 83.3% of hospital paediatrics. The answer ‘never’ is chosen in 35.29% of cases by free-lance paediatricians; the answer ‘often’, on the other hand, is given in 6.91% of cases by free-choice paediatricians. (Table 2; Fig. 1)

3. *The child has already been taken to other doctors*, *has already undergone numerous clinical examinations*, *but these have failed to identify the cause of the illness: a)never; b)sometimes; c) often.*

**Table 3:** Answers to question number 3

	a	b	c
Answers	24	318	166
%	4,72	62,60	32,68

Legend: a)never; b)sometimes; c) often

**Fig. 2:**
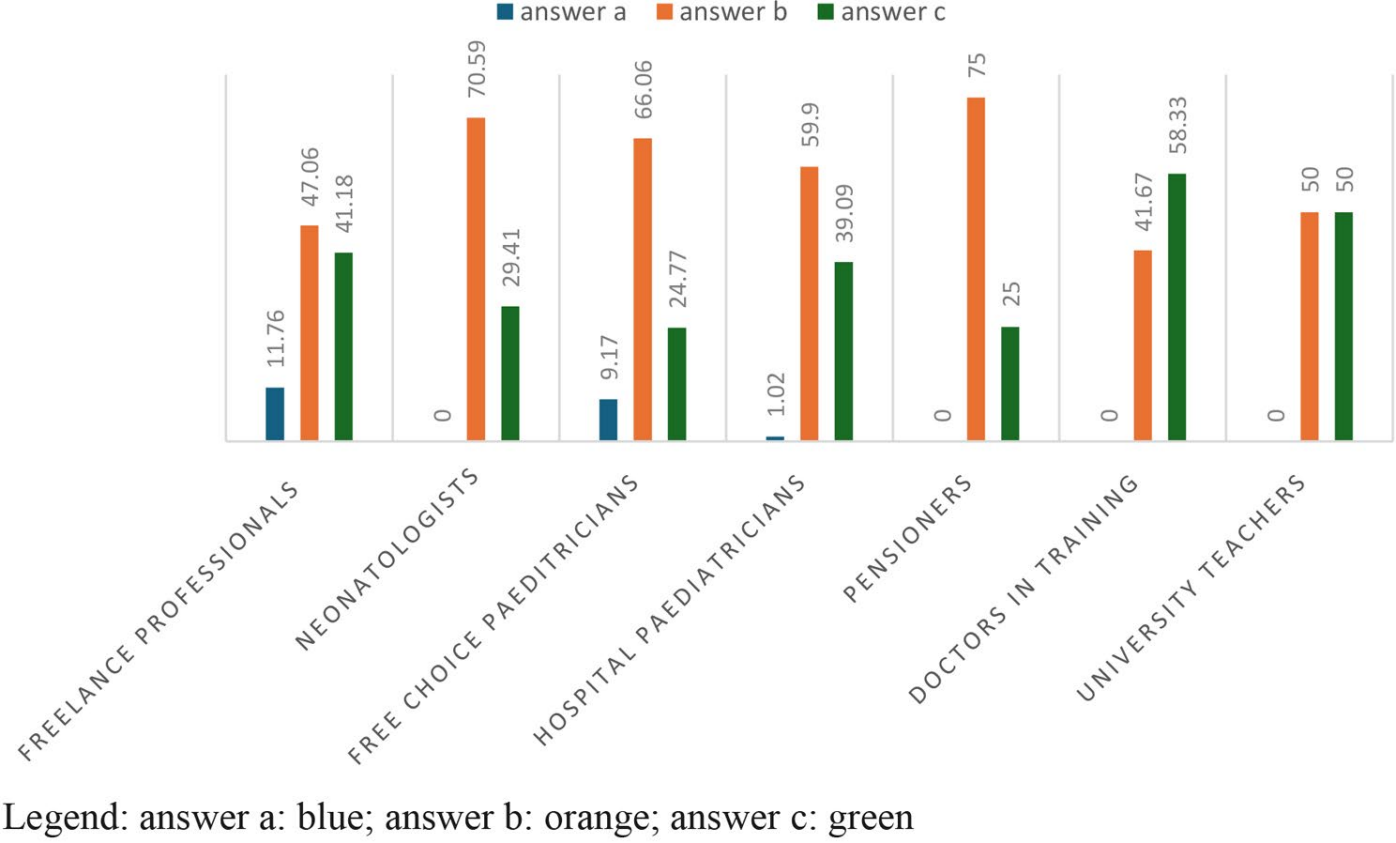
answers to question 3 divided according to profession

In Table 3 with a p-value = 0,0007885, 62.6% of the answers concerned the option “sometimes”, 66% of free choice paediatricians and 59.9% of hospital paediatricians giving this answer. The answer “never” is given more by freelance professionists in 11.76% of cases. University teachers answer 50% “sometimes”, and 50% “often”. (Table 3; Fig. 2)

4. *The child appears to be apparently healthy*, *but the parents claim that he/she has some kind of illness*, *proving it with analyses and/or medical records and/or opinions of other doctors: a)never; b) sometimes; c)often.*

**Table 4:** Answers to question number 4

	a	b	c
Answers	98	357	53
%	19,29	70,28	10,43

Legend: a)never; b)sometimes; c) often

**Fig. 3:**
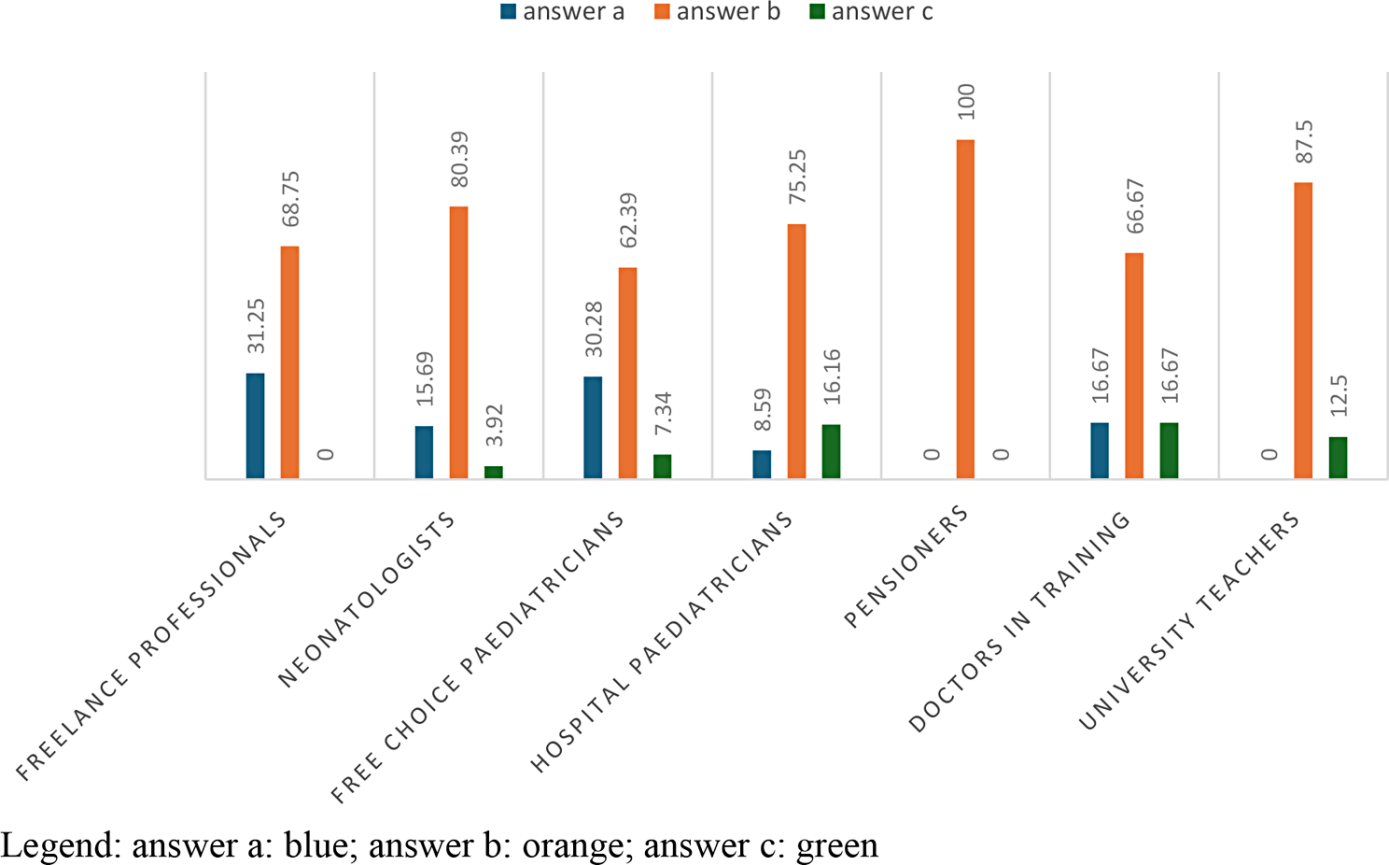
answers to question 4 C divided according to profession

In Table 4 with a p-value = 6.134e-06, 70.28% is ‘*sometimes*’, given in 80.39% of cases by neonatologists; in 75.25% of cases by hospital paediatricians and 87.50% of cases by university teachers. The answer “never” is chosen in 31.25% by freelance professionists and in 30.28% by paediatricians of free choice. 8Table 4, Fig. 3)

5. *Had these children been admitted to hospital before? Yes; no.*

**Table 5:** Answers to question number 5

	Yes	No
Answers	411	97
%	76,09	23,91

**Fig. 4:**
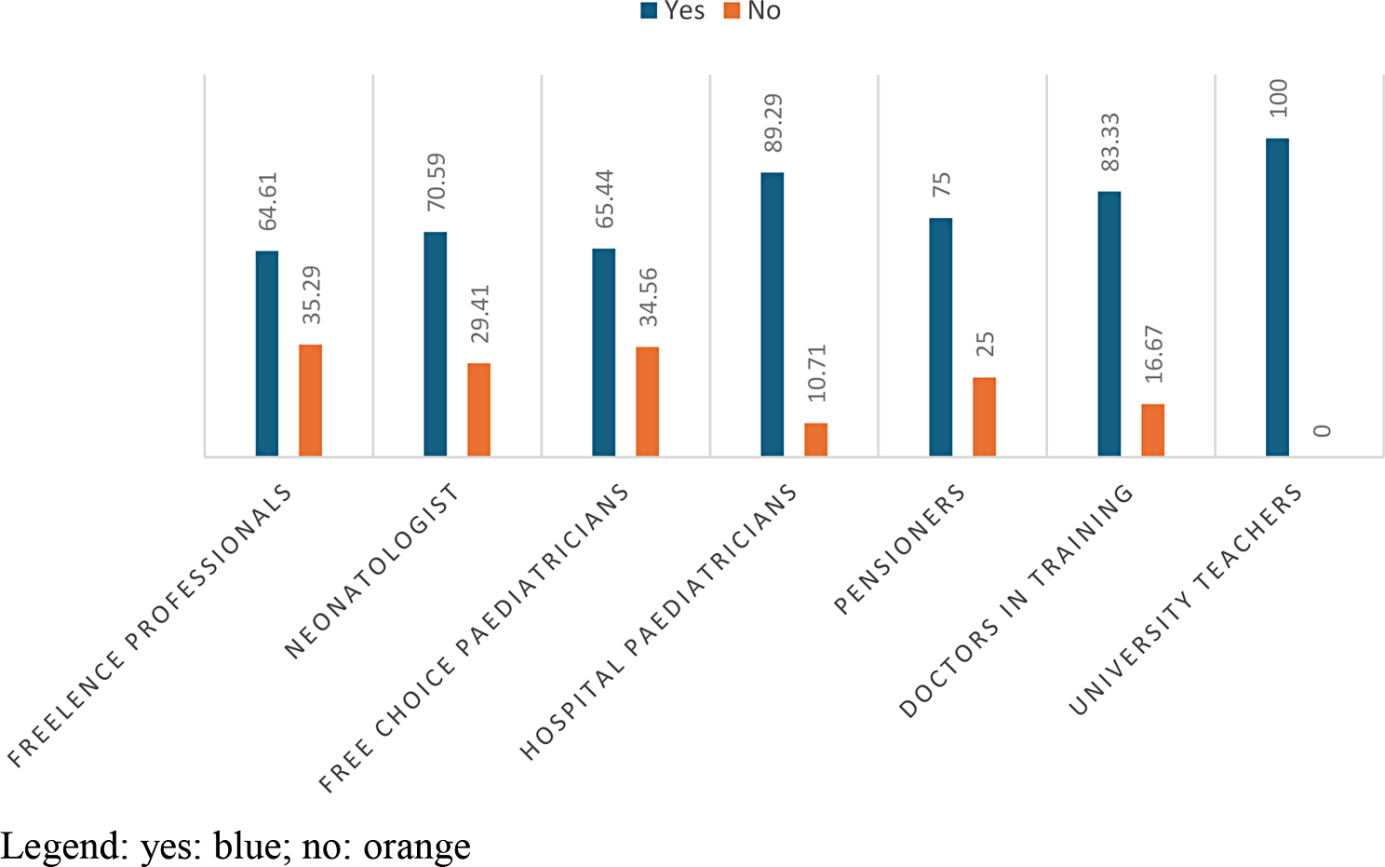
answers to question 5 divided according to profession

The answer “yes” is given in 76.09% of the cases and is given by 89.29% of the hospital paediatricians and 100% of the university paediatricians. The answer ‘no’ is given by 34.56% of free-choice paediatricians and 35.29% of freelancers. Therefore, by carrying out an analysis, the p-value that will emerge will be 1.493e-06. (Table 5; Fig. 4).

6. If the previous question (4 bis) is answered in the affermative, question 4 ter must also be answered: 4.(ter) If yes, how many? (a) One; (b) From one to five; (c) More than five.

**Table 6:** Answers to question number 6

	a	b	c
Answers	82	293	36
%	19,95	71,29	8,76

Legend: answer a: One; answer b: From one to five; answer c: More than five

The 71.29% of the professionals who answered the previous question (4 bis) with “Yes”, claim that the children have previously undergone between one and five hospitalizations.

**Fig. 5:**
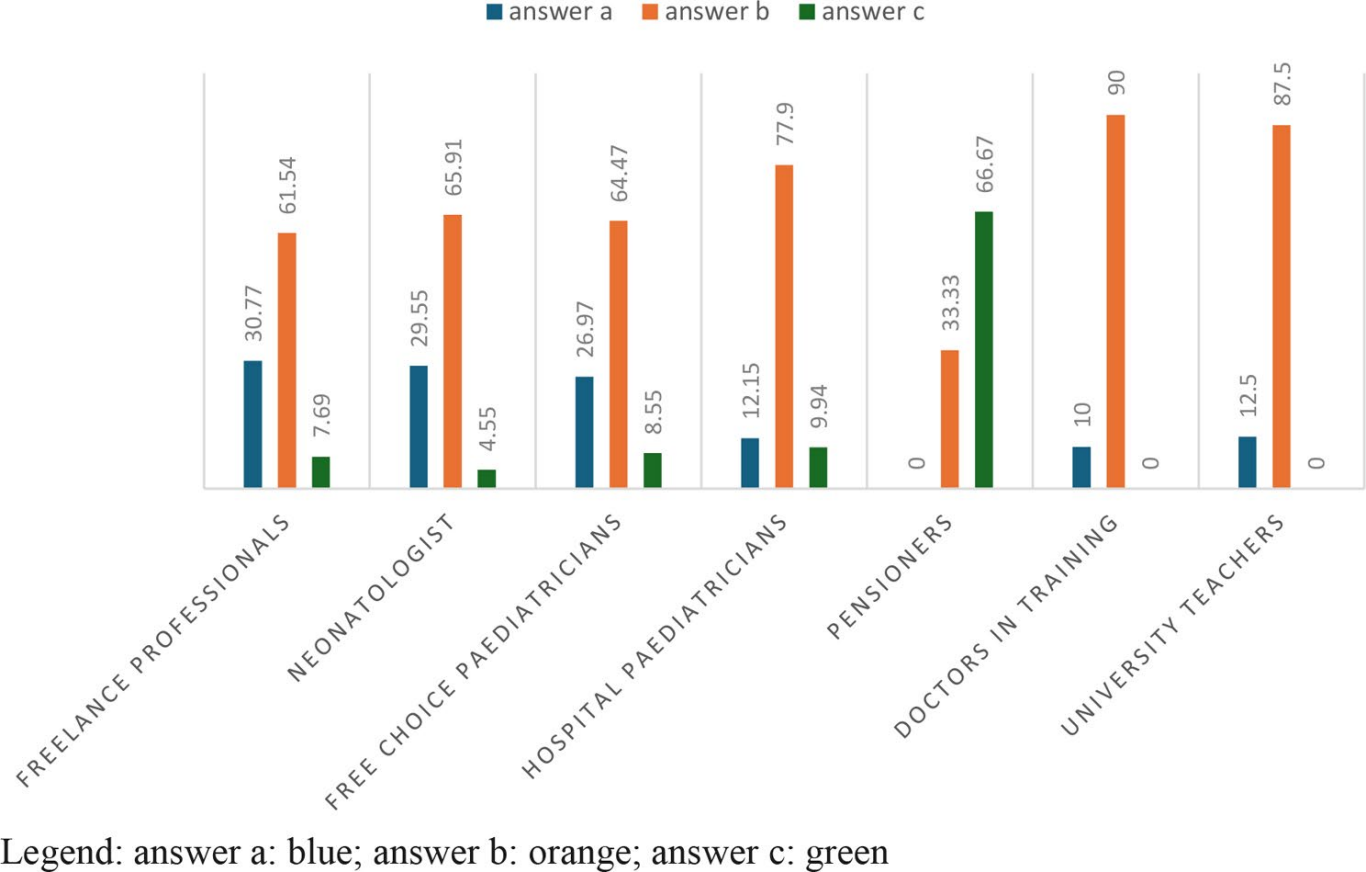
answers to the question 4 ter divided according to profession

In question 4 ter with a p-value = 0.001655, it is highlighted that for hospital paediatricians 77.9%, and for specialists with 90%, children have undergone from one to five hospitalizations, while freelancers with 30.77% and paediatricians of free choice with 26.97% instead support the response to (only one hospitalization). This also highlights the differences between intra- and extra-hospital settings. (Table 6; Fig. 5)

7. *The behaviour of these parents during the child’s visits was: normal; particularly anxious; calm*, *but they recounted in detail the previous examinations*, *the medical examinations carried out*, *the hospitalisations undergone by the child.*

**Table 7:** Answers to question number 7

	a	b	c
Answers	30	249	222
%	5,99	49,70	44,31

Legend: a: normal; b: anxious; c: quiet

To this question, 49.70% of health professionlas answered: Anxious; 44.31% of health workers answer Quiet.

**Fig. 6:**
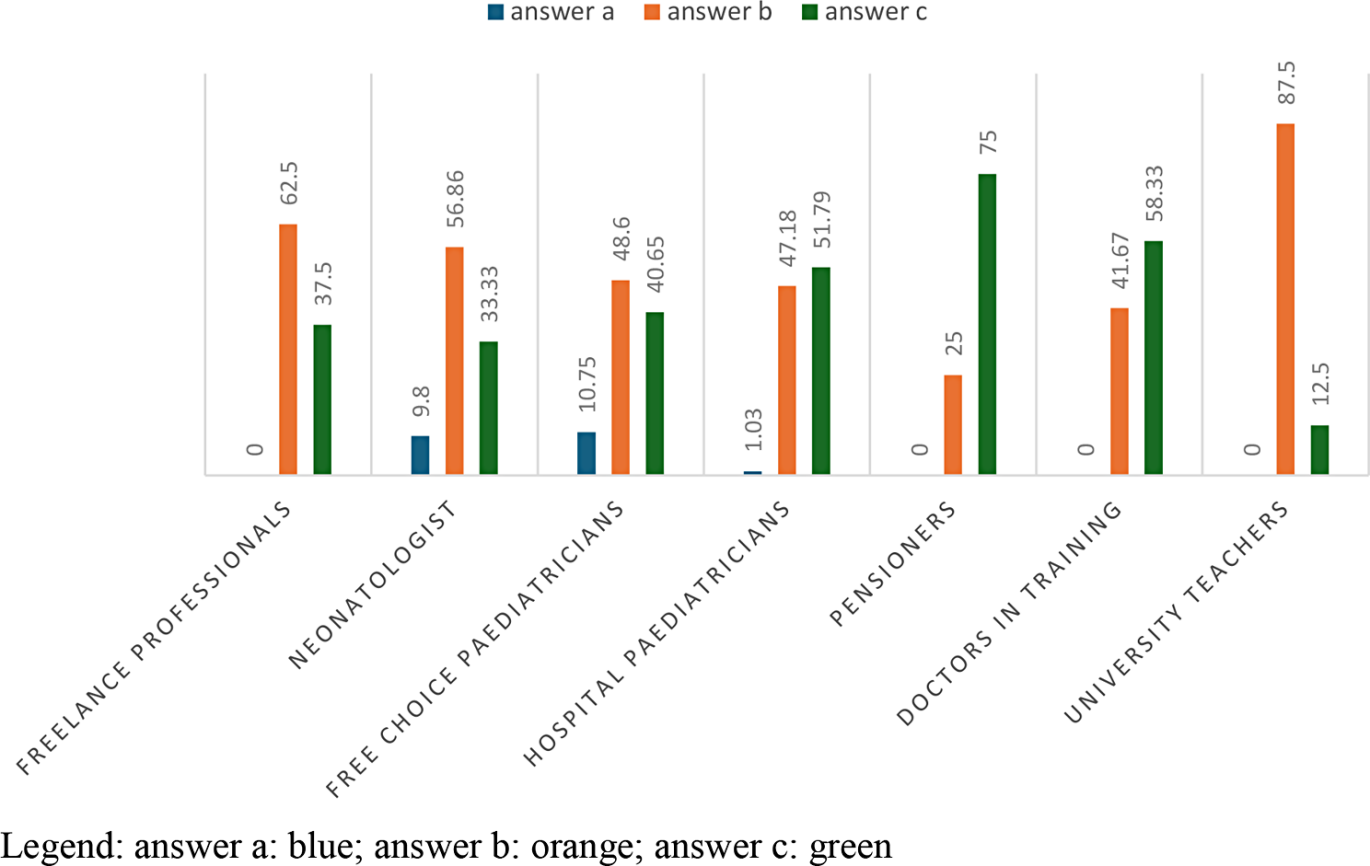
answers to question 7 divided according to profession

In Figs. 6 and 62.40% of freelancers, 48.60% of self-employed paediatricians and 87.50% of university teaching provide the answer “Anxious” (answer b). The “quiet” option (answer c) is chosen by 51.79% of hospital paediatricians and by 58.33% of residents. Therefore, analyzing the various answers with a p-value = 0.001444, we will always notice this slight discrepancy between the perception of those who work in a hospital environment and those who do not. (Table 7; Fig. 6)

8. *Among the parents of the type described above*,* have you ever discovered lies hidden by them about the child’s health or mystifications such as: Induced provocation of disease symptoms; alteration of analyses and/or medical records; administration of non-prescribed drugs or different doses than prescribed; administration of commonly used substances (e.g. salt*,* water*,* sugar) in exaggerated quantities*,* such that they could still cause harm to the child.*

**Table 8:** Answers to question number 8

	a	b	c
Answers	238	36	63
%	70,62	10,68	18,69

Legend: in 70.62% of cases health professionals answered “symptom provocation”

**Fig. 7:**
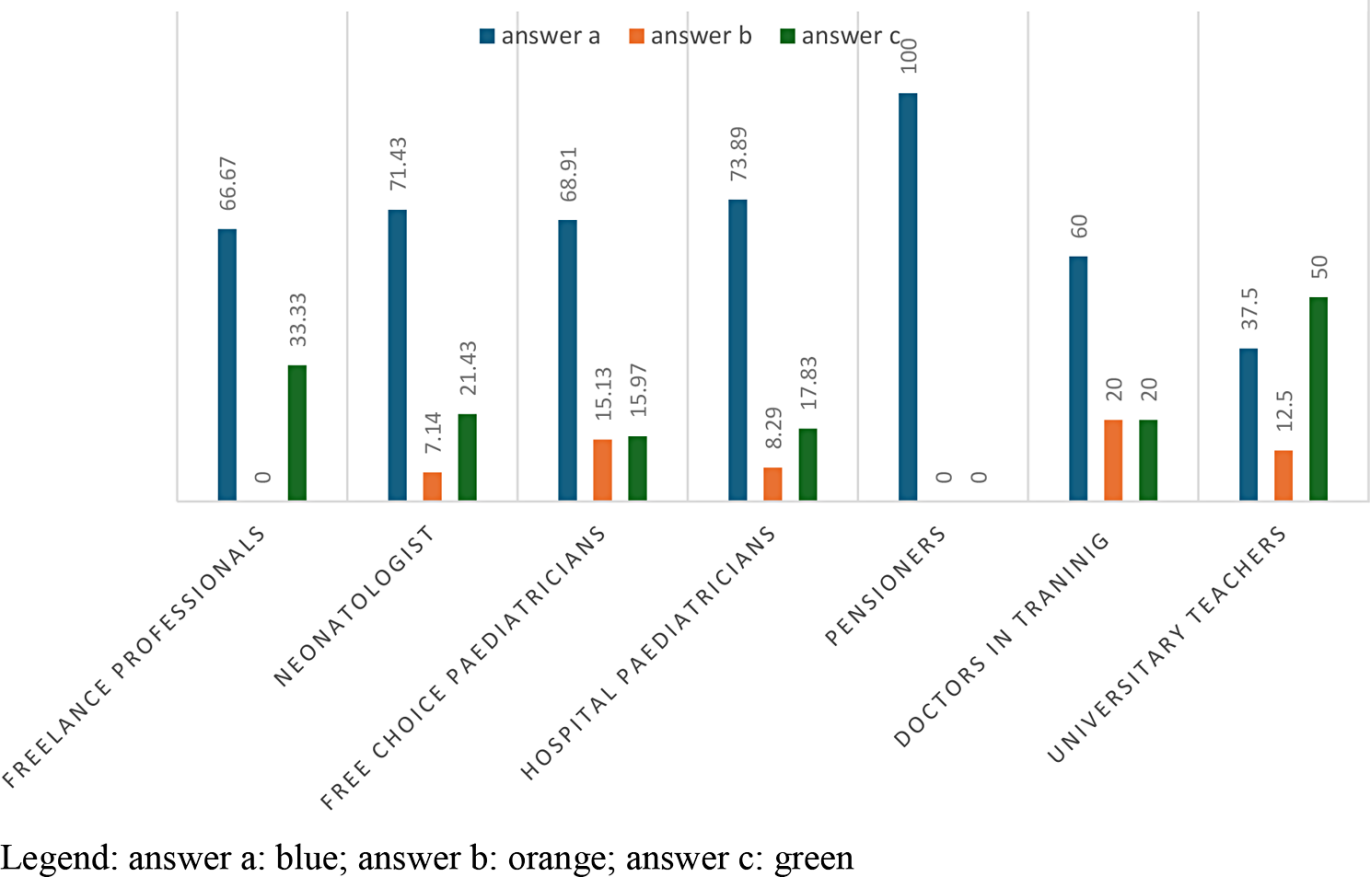
answers to question 8 divided according profession

The answer most frequently given by health professionals in 70.62% of cases is the first one, i.e. symptom provocation. And there are no particular differences between the various categories of professionals. (Table 8; Fig. 7)

9. *Have you ever observed a worsening of symptoms upon discharge? A) never; b) sometimes; c)often.*

**Table 9:** Answers to question number 9

	a	b	c
Answers	127	300	60
%	26,08	61,60	12,32

Legend: A) never; b) sometimes; c)often

**Fig. 8:**
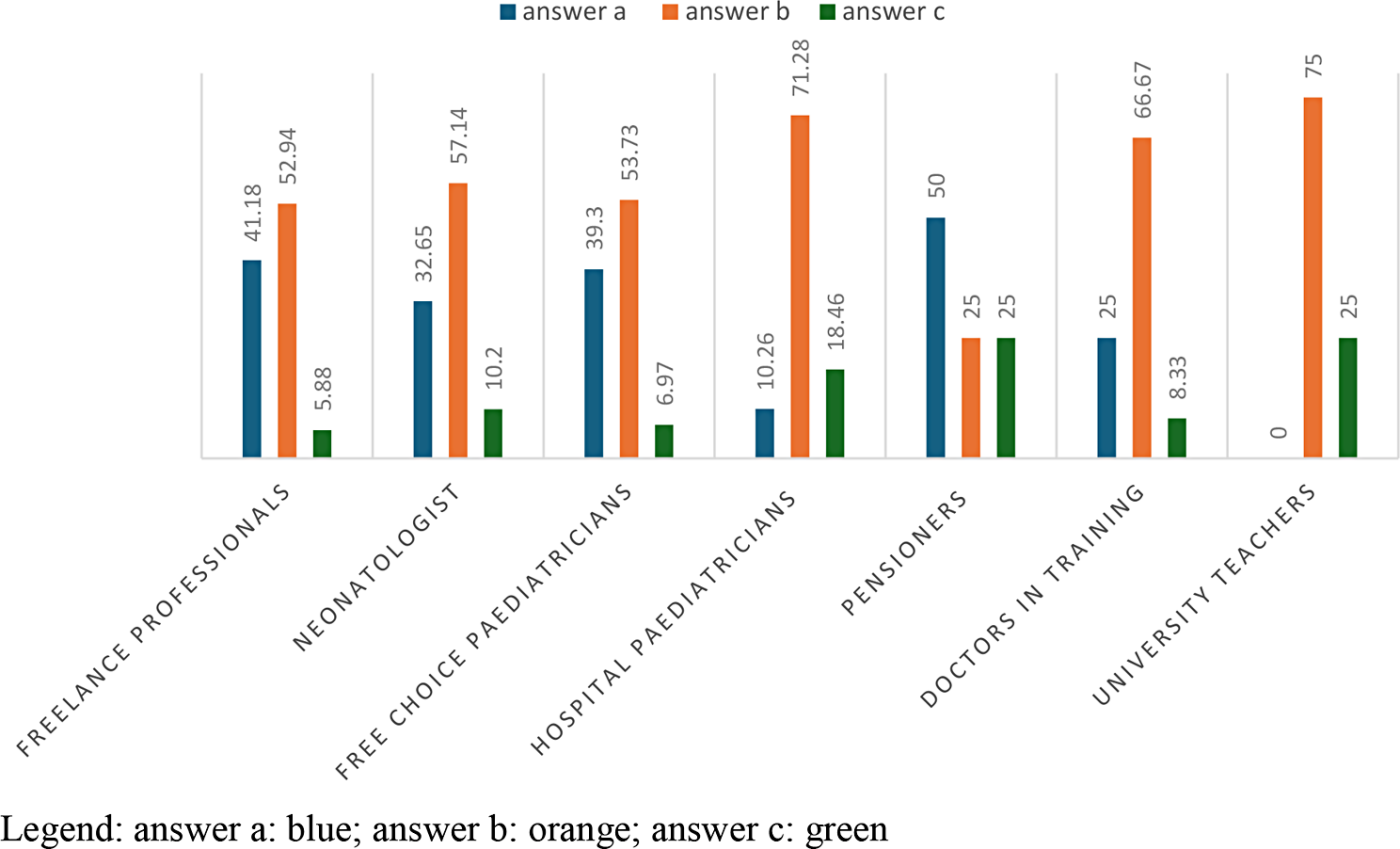
answers to question 9 divided according to profession

In Tables 9, 61.60% of the answers from health professionals are ‘sometimes’, and the majority of answers are given by academics (75%) and hospital paediatricians (71.28%). 41.18% of freelancers and 39.30% of paediatricians of free choice answered ‘never’. (Table 9; Fig. 8)

10. *In the cases described above*, *what did you do? You have asked for an interview with the parents; you have referred the parents to a specialised colleague (psychiatrist*, *psychologist); you have reported the situation to the social services; you have reported the case to the judicial authorities.*

**Table 10:** Answers to question number 10

	a	b	c	d
Answers	180	201	64	12
%	39,39	43,98	14	2,63

Legend: answer a: You have asked for an interview with the parents; answer b: you have referred the parents to a specialised colleague (psychiatrist, psychologist); answer c: you have reported the situation to the social services; answer d: you have reported the case to the judicial authorities

43% of health *professionals answered ‘you have referred the parents to a specialised colleague (psychiatrist*, *psychologist)’;* 39.39% of health professionals answered ‘*you have asked for an interview with the parents’;* 14% of health professionals answered ‘*you have reported the situation to the social services’* and finally 2.63% of health professionals answered ‘*you have reported the case to the judicial authority’.*

**Fig. 9:**
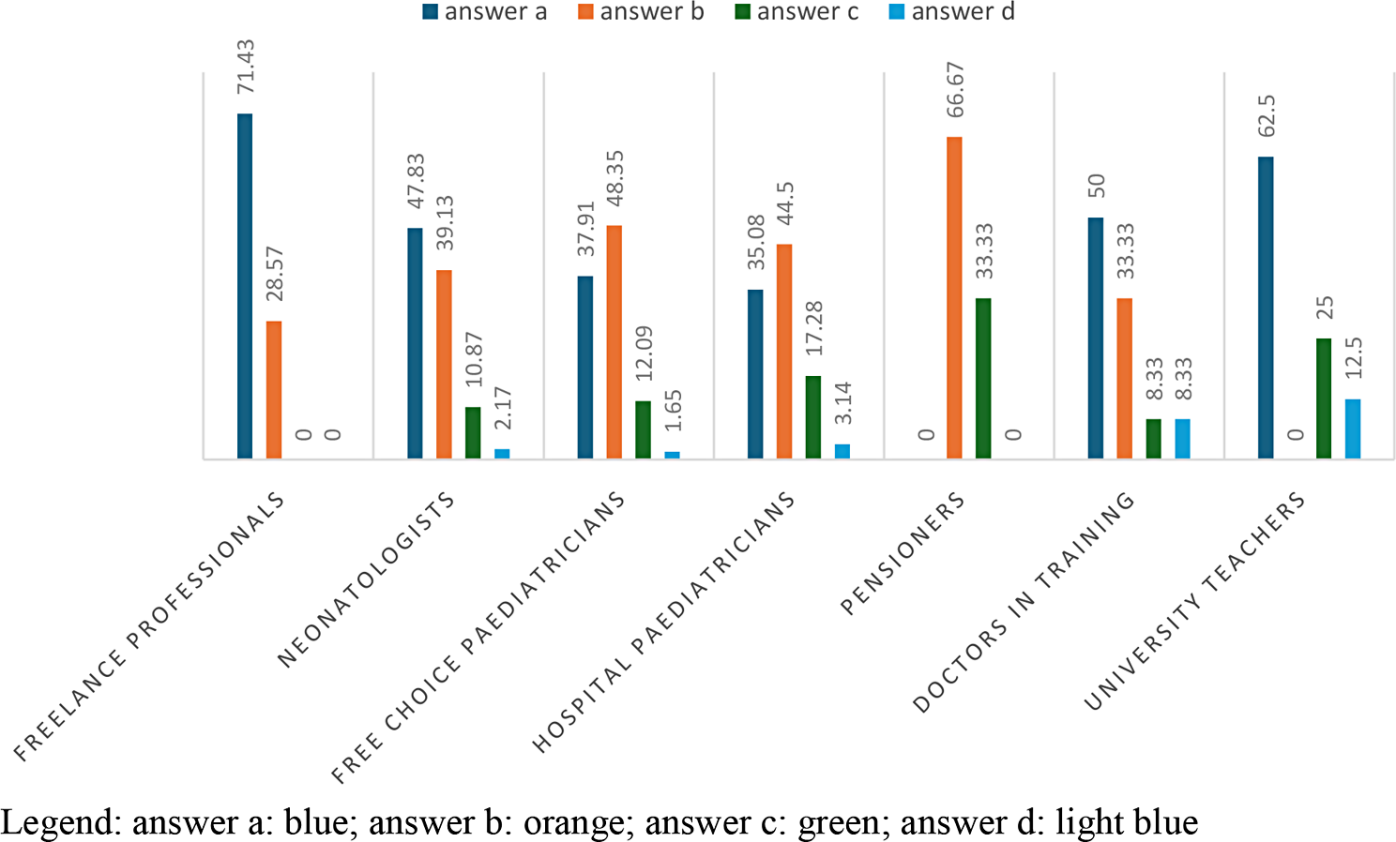
answers to question 10 divided according to profession

1. 71.43% of the freelancers requested an interview with the parents and in no case reported the case to the judicial authority or social services.
2. 48.35% of free-choice paediatricians requested an interview with medical specialists (psychiatrist, psychologist); 37.91% of free-choice paediatricians requested an interview with parents; 12.09% of free-choice paediatricians proceeded to report the case to the social services and only 1.65% of free-choice paediatricians reported the case to the judicial authorities.
3. 44.50% of hospital paediatricians request an interview with medical specialists; 35.08% of hospital paediatricians request an interview with parents; 17.28% proceed to report the case to the social services and 3.14% of hospital paediatricians report the case to the judicial authorities. (Table 10; Fig. 9).

Analyzing the difference between male professionals and female professionals: with a p-value = 0.02896, we will notice a discrepancy in the type of attitude implemented, the professional women will tend more to interview the parents in 41.28% of cases, the specialist male will instead tend to report more to the social services with a 22.52%.

11. *Have you ever heard of Munchausen syndrome by proxy? Yes; no.*

**Table 11:** Answers to question number 11

	a	b
Answers	492	13
%	97	3

Legend: answer a: yes; answer b: no

This result evidences that only 3% of professionists never heard to speak the term “Munchausen Syndrome”.

**Fig. 10:**
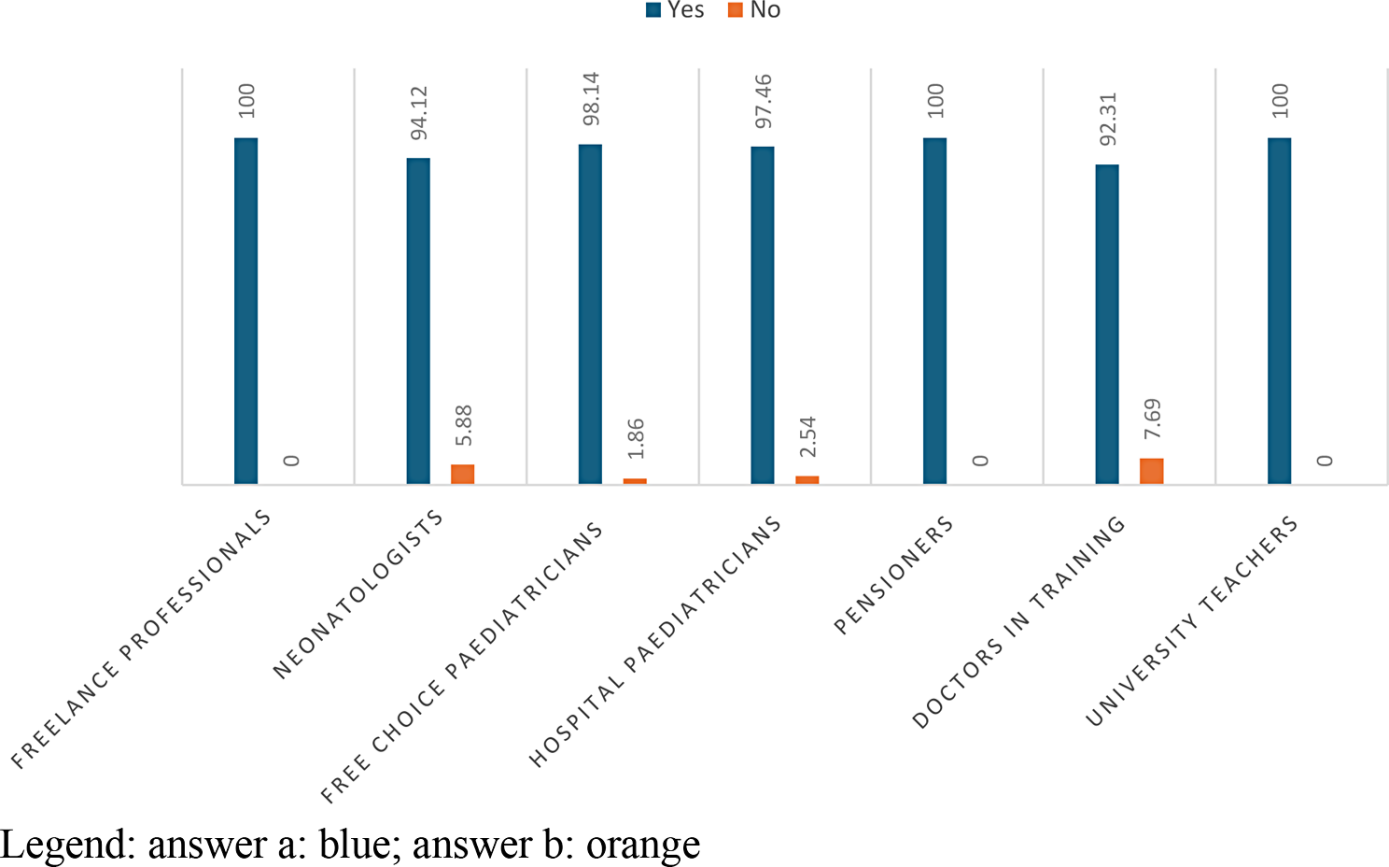
answers to question 11 divided according to professional profiles

Analyzing the details of results of question number 11 we observe that 97.43% of health professionals answered in the affermative and 2.57% answered negatively. The 98.14% of free-choice paediatricians and 97.46% of hospital paediatricians answered in the affermative. Most neonatologists (5.88%) and doctor in training (7.69%) are not aware of Munchausen’s syndrome. (Table 11; Fig. 10).

## Discussion

Munchausen Syndrome by Proxy (MSBP) poses significant challenges to healthcare professionals due to its elusive nature and potential harm to victims. Despite its recognition for many years, the medical literature lacks consistent studies on its epidemiology, therapeutic approaches, and prognosis. This article aims to shed light on the critical importance of early diagnosis, effective management, and appropriate reporting to protect the welfare of those affected by MSBP.

The study conducted underlines that there are some discrepancies in response between the various professions, especially sectoral between those who work in hospitals and those who do not. Example, in question 4 A we notice a discrepancy between the answers and the perceptions that freelancers and hospital paediatricians have. Most doctors know Münchausen Syndrome by proxy. Finally, when there is a strong suspicion of the syndrome, however, in most cases a discussion is sought with the parent or with the specialist instead of referring to the competent authorities.

Recognizing and diagnosing MSBP in a clinical setting is fundamental for preventing further abuse and minimizing unnecessary medical or surgical interventions. When a case of MSBP is suspected, medical professionals must prioritize arriving at a clinical diagnosis. A comprehensive clinical examination of the child and an in-depth analysis of the family’s psycho-social relationship should be thoroughly conducted and documented. Healthcare professionals face complex ethical dilemmas in managing cases of MSBP. Balancing the child’s best interest with the principle of substituted judgment adds to the intricacy of decision-making. In such situations, doctors are called upon to collaborate with other professionals and law enforcement representatives when necessary, adhering to the Italian Deontological Medical Code (Article 32) to ensure total care of the vulnerable person.

**Fig. 11:**
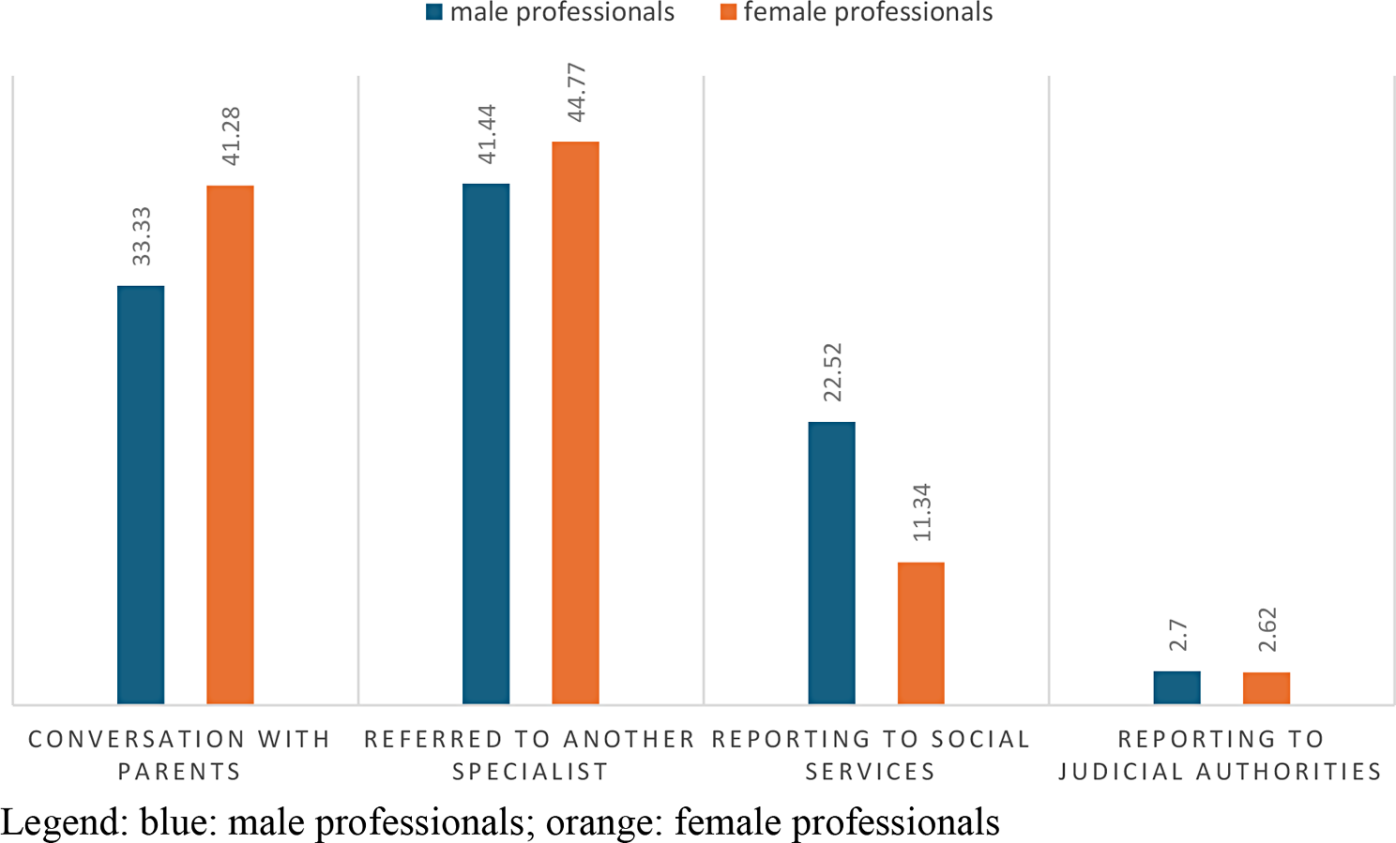
answers to question 10 divided according to gender

From the results obtained from the administered questionnaire, it emerged that only 2% of pediatric professionals turned to the competent judicial authority through a mandatory report. This data requires many reflections. (Table 10; Figs. 9 and 11)

Prompt reporting of known or suspected cases of child abuse and neglect is a legal and ethical obligation for medical professionals [18–20]. Reporting duties have been enacted in child protection legislation in some jurisdictions, while organizational policies govern reporting in others. Training interventions should be employed to equip professionals with the knowledge, awareness, and attitudes required for effective reporting. Well-trained interdisciplinary child protection teams, using an evidence-based approach, play a crucial role in accurately identifying cases of maltreatment and making decisions that positively impact the well-being of affected children. Public policies, such as mandatory reporting laws, contribute to tertiary prevention interventions aimed at safeguarding maltreated children [21–25].

A very recent study published in the journal Child Abuse & Neglect highlighted through the description of a group of patients aged between 10 and 18 that the victims had had a major impact on the quality of their lives with a high rate of school dropout (96%). The average delay to the suspected diagnosis was 5.8 (2.6–9) years and, even when recognised, Munchausen syndrome by proxy was rarely the subject of social or judicial reporting (only in 42% of adolescents) [26].

It is important to highlight the limitations of this study; firstly, the answers in fact refer to a relatively limited number of paediatricians; furthermore, a training course was not implemented following the administration of the questionnaire, on the areas of uncertainty and lack of knowledge of health care professionals; however, the interest of this subject and duties related both to patients and referral to law authorities encourage to improve the guidelines and protocols to be applied in “ad hoc” hospital care pathways for suspected case of MBP.

## Conclusions

We emphasize the urgency of enhancing the recognition and management of Munchausen Syndrome by Proxy. Early diagnosis, appropriate reporting, and collaboration with child protection authorities are essential in safeguarding the well-being of vulnerable individuals. Emphasizing the role of interdisciplinary teams and implementing evidence-based interventions is crucial for protecting the most marginalized members of society—maltreated children.

## Data Availability

The datasets used and analyzed during the current study are available from the corresponding author on reasonable request.

## Abbreviations

MBP: Munchausen Syndrome by Proxy

## References

[1] Asher R. Munchausen’s syndrome. Lancet. 1951;6650:339–41. 10.1016/s0140-6736(51)92313-6 14805062

[2] Sousa Filho D, Kanomata EY, Feldman RJ, et al. Munchausen syndrome and Munchausen syndrome by proxy: a narrative review. Einstein (Sao Paulo). 2017 Oct-Dec;15(4):516–21. 10.1590/S1679-45082017MD3746 PMC5875173 29364370

[3] Thomas K. Munchausen Syndrome by Proxy: identification and diagnosis. J Pediatr Nurs. 2003;8(3):174–80. 10.1053/jpdn.2003.35 12796859

[4] Unal EO, Unal V, Gul A et al. A serial Munchausen Syndrome by Proxy. Indian J Psychol Med 2017 Sep-Oct; 39(5):671–4. 10.4103/0253-7176.217017 PMC5688899 29200568

[5] Anderson APA, Feldman MD, Bryce J. Munchausen by Proxy: a qualitative investigation into online perceptions of medical child abuse. J Forensic Sci. 2018;63(3):771–5. 28766877 10.1111/1556-4029.13610

[6] Rini MS, Colucci C, Bucci MB, et al. Child abuse hidden in plain sight: the dentist obligations. Dent Cadmos. 2017;85(10):647–56.

[7] Kuhne ACA, Pitta AC, Galassi SC, et al. Munchausen by proxy syndrome mimicking childhood-onset systemic lupus erythematosus. Lupus. 2019;28(2):249–52. 30616452 10.1177/0961203318821156

[8] Lanzarone A, Nardello R, Conti E et al. Child abuse in a medical setting: case illustrations of two variants of munchausen sindrome by proxy. EMBJ 2017 March 12 (10): 047–50.

[9] Takher AS, Cosme RM. Unmasking the Enigma: a Case Report of Catatonia unveiled as Munchausen by Proxy. Cureus. 2023;15(8):e43082. 37680395 10.7759/cureus.43082 PMC10482309

[10] Alkhattabi F, Bamogaddam I, Alsagheir A, et al. Munchausen syndrome by proxy: a case report. J Med Case Rep. 2023;17(1):148. 37013583 10.1186/s13256-023-03848-7 PMC10071623

[11] Bertrand V, Millardet E, Bouchereau J, et al. Suspicion of Munchausen syndrome by proxy with a child’s presentation of undernutrition, scurvy, and an apparent avoidant restrictive food intake disorder. Eat Weight Disord. 2022;27(8):3815–20. 36565378 10.1007/s40519-022-01520-5

[12] Abdurrachid N, Gama Marques J. Munchausen syndrome by proxy (MSBP): a review regarding perpetrators of factitious disorder imposed on another (FDIA). CNS Spectr. 2022;27(1):16–26. 32772954 10.1017/S1092852920001741

[13] Pasqualone GA, Fitzgerald SM. Munchausen by proxy syndrome: the forensic challenge of recognition, diagnosis, and reporting. Crit Care Nurs Q. 1999;22(1):52–64. 10646463 10.1097/00002727-199905000-00007

[14] Sagot AJ, Weiss KJ. Preserving immnity for reporters of medical child abuse. J Am Acad Psychiatry Law. 2022;50(4):618–25. 36223940 10.29158/JAAPL.220030-21

[15] Çakmaklı HF, Ertem M, Ünal İnce E, et al. A medical child abuse case with spurious bleeding; importance of collecting the evidence. J Forensic Leg Med. 2024;107:102741. 39208469 10.1016/j.jflm.2024.102741

[16] Sheridan MS. The deceitcontinues: An updated literature review of MunchausenSyndrome by Proxy, in *Child Abuse and Neglect*, Apr. 2003, vol. 27, no. 4, pp. 431–451. 10.1016/S0145-2134(03)00030-9 10.1016/s0145-2134(03)00030-9 12686328

[17] Jenny C, Metz JB. Medical child abuse and medical neglect. Pediatr Rev. 2020;41(2):49–60. 32005682 10.1542/pir.2017-0302

[18] Bartsch C, Risse M, Schütz H, et al. Munchausen syndrome by proxy (MSBP): an extreme form of child abuse with a special forensic challenge. Forensic Sci Int. 2003;137(2–3):147–51. 14609650 10.1016/j.forsciint.2003.07.007

[19] Bursch B, Emerson ND, Sanders MJ. Evaluation and management of factitious disorder imposed on another. J Clin Psychol Med Settings. 2021;28(1):67–77. 31612305 10.1007/s10880-019-09668-6

[20] Alwan RM, Atigapramoj NS. Child maltreatment and neglect. Emerg Med Clin North Am. 2021;39(3):589–603. 34215404 10.1016/j.emc.2021.04.009

[21] Gehlawat P, Gehlawat VK, Singh P, et al. Munchausen syndrome by proxy: an alarming face of child abuse. Indian J Psychol Med. 2015;37(1):90–2. 25722520 10.4103/0253-7176.150850 PMC4341319

[22] Flaherty EG, MacMillan HL, Christian CW, et al. Caregiver-fabricated illness in a child: a manifestation of child maltreatment. Pediatrics. 2013;132(3):590–7. 23979088 10.1542/peds.2013-2045

[23] Ozdemir DF, Yalçın SS, Akgül S, et al. Munchausen by proxy syndrome: a case series study from Turkey. J Fam Viol. 2015;30(5):661–71.

[24] Braham MY, Jedidi M, Chkirbene Y, et al. Caregiver-fabricated illness in a child: a case report of three siblings. J Forensic Nurs. 2017;13(1):39–42. 28212199 10.1097/JFN.0000000000000141

[25] Giurgea I, Ulinski T, Touati G, et al. Factitious hyperinsulinism leading to pancreatectomy: severe forms of Munchausen syndrome by proxy. Pediatrics. 2005;116(1):e145–8. 15995015 10.1542/peds.2004-2331

[26] Abraham-Bizot A, Greco C, Quartier P, et al. Medical child abuse: medical history and red flags in French adolescents. Child Abuse Negl. 2023;146:106523. 37950944 10.1016/j.chiabu.2023.106523

